# Highly Sensitive and Selective Potassium Ion Detection Based on Graphene Hall Effect Biosensors

**DOI:** 10.3390/ma11030399

**Published:** 2018-03-07

**Authors:** Xiangqi Liu, Chen Ye, Xiaoqing Li, Naiyuan Cui, Tianzhun Wu, Shiyu Du, Qiuping Wei, Li Fu, Jiancheng Yin, Cheng-Te Lin

**Affiliations:** 1 Faculty of Materials Science and Engineering, Kunming University of Science and Technology, Kunming 650093, China; liuxiangqi@nimte.ac.cn; 2 Key Laboratory of Marine Materials and Related Technologies, Zhejiang Key Laboratory of Marine Materials and Protective Technologies, Ningbo Institute of Materials Technology and Engineering (NIMTE), Chinese Academy of Sciences, Ningbo 315201, China; yechen@nimte.ac.cn (C.Y.); lixiaoqing@nimte.ac.cn (X.L.); yuanyuanpenny@stu.xjtu.edu.cn (N.C.); 3 College of Material Science and Optoelectronic Technology, University of Chinese Academy of Sciences, 19 A Yuquan Rd., Shijingshan District, Beijing 100049, China; 4 MOE Key Laboratory for Non-Equilibrium Synthesis and Modulation of Condensed Matter, Xi’an Jiaotong University, Xi’an 710049, China; 5 Shenzhen Institutes of Advanced Technology, Chinece Acedemy of Science, Shenzhen 518055, China; tz.wu@siat.ac.cn; 6 Ningbo Institute of Materials Technology and Engineering, Chinese Academy of Sciences, Ningbo 315201, China; dushiyu@nimte.ac.cn; 7 School of Materials Science and Engineering, Central South University, Changsha 410083, China; qiupwei@csu.edu.cn; 8 College of Materials and Environmental Engineering, Hangzhou Dianzi University, Hangzhou 310018, China; fuli@hdu.edu.cn

**Keywords:** single-layer graphene, Hall effect biosensor, guanine-rich DNA strand, guanine-quadruplexes, potassium ions

## Abstract

Potassium (K^+^) ion is an important biological substance in the human body and plays a critical role in the maintenance of transmembrane potential and hormone secretion. Several detection techniques, including fluorescent, electrochemical, and electrical methods, have been extensively investigated to selectively recognize K^+^ ions. In this work, a highly sensitive and selective biosensor based on single-layer graphene has been developed for K^+^ ion detection under Van der Pauw measurement configuration. With pre-immobilization of guanine-rich DNA on the graphene surface, the graphene devices exhibit a very low limit of detection (≈1 nM) with a dynamic range of 1 nM–10 μM and excellent K^+^ ion specificity against other alkali cations, such as Na^+^ ions. The origin of K^+^ ion selectivity can be attributed to the fact that the formation of guanine-quadruplexes from guanine-rich DNA has a strong affinity for capturing K^+^ ions. The graphene-based biosensors with improved sensing performance for K^+^ ion recognition can be applied to health monitoring and early disease diagnosis.

## 1. Introduction

Potassium (K^+^) ion is predominantly an intracellular cation in biological systems [1,2], and is involved in various physiological and pathological events, including enzyme activation, nervous transmission, blood pressure/pH regulation, membrane potential modulation in living cells, etc. [3,4]. Many diseases, including alcoholism, anorexia, bulimia, diabetes, and heart disease, have been demonstrated to be significantly related to the imbalance of potassium ion concentration [5]. Moreover, due to the fact that the concentration of K^+^ ions (≈150 mM) inside the cells of the human body is over 30 times higher than that in the extracellular fluid [6], the abnormal K^+^ ion concentrations in the extracellular matrix of tumors would lead to the suppression of immune responses [7]. In order to identify K^+^ ions, different approaches such as fluorescent [8,9], colorimetric [10,11], electrochemical [12], and electrical detection methods [13] using a variety of nanomaterials have been widely investigated. Zeng et al. fabricated an electrochemical transducer based on hydrothermal synthesized MoS_2_ nanoflowers that had a detection limit of ≈3.2 μM for determining K^+^ ions [14]. Lu et al. synthesized Fe_3_O_4_/C core-shell nanoparticles grafted with guanine-rich oligonucleotides as a fluorescent sensing platform, which exhibited high sensitivity as low as 1.3 μM for K^+^ ion analysis [15]. However, in previous reports, the limited selectivity against sodium ions and the low detection sensitivity (commonly ≈μM) may restrict their clinical applications. Therefore, it is of great importance and is a significant challenge to develop a nanobiosensor for highly sensitive and selective detection of K^+^ ions in aqueous environments. Graphene-based biosensors have attracted much research interest recently.

Due to its atomically thin nature, good biomolecular compatibility, and exceptional electrical properties [16,17,18], graphene has been extensively studied as a promising nanomaterial for biosensing applications [19]. Nowadays, a variety of nanobiosensors constructed with graphene have been implemented for the recognition of biomolecules with high sensitivity and specificity, such as ions [20], glucose [21], dopamine [22], deoxyribonucleic acid (DNA) [23], etc. Electrolyte-gated field-effect transistors (FETs) fabricated with mechanically exfoliated graphene have been demonstrated to achieve an ultralow detection limit of 10 nM for sensing K^+^ ions [24]. However, using mechanical exfoliation, the lateral size of the samples is usually in micrometer scale and their thickness is randomly distributed, thus limiting the practical applications. In contrast, high-quality single-layer graphene films can be prepared over wafer-scale areas by a catalytic growth technique of chemical vapor deposition (CVD) [25,26]. Compared to its derivatives (e.g., graphene oxide and mechanically exfoliated graphene), the graphene grown by CVD has inherent advantages for the fabrication of biosensing devices, because the layer number can be easily controlled and the electrical properties are more uniform over a large area [27]. Moreover, label-free electrical detection based on graphene has attracted significant academic attention in recent years due to the low cost-in-use, process simplicity, and non use of fluorescent labels [28]. Li et al. reported that the K^+^ ion-sensitive FETs based on CVD-grown graphene exhibited good performance with a detection limit of 1 μM, which is comparable to commercial silicon sensors [13]. As a result, there is high demand for the exploration of the potential of CVD graphene biosensors for label-free recognition of K^+^ ions with ultralow detection limit, as well as high selectivity against sodium.

In this contribution, we fabricated the Hall effect biosensors made of CVD-grown single-layer graphene for detecting K^+^ ion. Distinct from the two-terminal resistor and FET methods, the sensor measurements based on the Van der Pauw technique are able to monitor the multiple electrical properties of graphene films during the detection. The Van der Pauw method is usually employed to determine the sheet resistance and the Hall coefficients (carrier concentration/mobility) of a thin semiconductor, by placing it in a magnetic field with a four-point electrode configuration [23]. The carrier concentration is defined as the number of charge carriers in a given area, and the mobility characterizes how fast the carriers can migrate through a material. Our K^+^ ion biosensors exhibit a high sensitivity as low as 1 nM (10^−9^ M) and an excellent selectivity (≈4 times) for effectively distinguishing between potassium and sodium ions. The results presented here provide helpful guidance for the design of high-performance ion-selective biosensors for use in healthcare and medical monitoring applications.

## 2. Materials and Methods

The graphene films were grown using a thermal CVD with the employment of a 25 µm-thick copper foil as the catalyst (Alfa Aesar, Haverhill, MA, USA, No. 13382, purity: 99.8%) with 25 µm in thickness. The pristine copper foil was cleaned in acetone by ultrasonication for 10 min to remove surface-adsorbed organic impurities. In order to grow graphene, copper foil was set in a tube furnace system, which was heated from room temperature to 1050 °C at a heating rate of 17.5 °C/min with 40 sccm hydrogen flow. When the furnace temperature reached 1050 °C, the copper foil was annealed for an additional 40 min for full reduction of native surface oxide layer. A gas mixture of CH_4_ and H_2_ (15:15 sccm) was then flowed into the system to form graphene layers on the copper surface at 1050 °C for reaction of 20 min, followed by cooling the furnace down naturally to room temperature.

In order to fabricate the biosensor, the as-prepared graphene films need to be detached from copper foil and then placed on the silicon substrate coated with a 300 nm insulating SiO_2_ layer. The copper foil with graphene layers on the surface was first cut into a sheet with a size of 8 mm × 8 mm, and then a ≈200 nm-thick PMMA layer was deposited on the graphene surface by spin-coating at a spin speed of 500 rpm (10 s) and 4000 rpm (1 min), respectively. The copper foil was then etched away by immersing the sample in 0.1 M ammonium persulfate ((NH_4_)_2_S_2_O_8_) aqueous solution at 60 °C for 5 h. After complete removal of copper, the PMMA/graphene layer was cleaned with deionized water 3 times and fished onto the SiO_2_/Si substrate. The sample was dried at 60 °C for 30 min on the hot plate, followed by immersing in acetone at 60 °C for 6 min to dissolve the PMMA. Finally, a post-annealing treatment was performed at 450 °C to improve the interface between graphene and the substrate, as well as guarantee the surface cleanness by decomposition of PMMA micro-residues.

The morphology, quality, and surface chemical compositions of graphene films were identified using optical microscope (OM, LSM700, Zeiss, Oberkochen, Germany), atomic force microscopy (AFM, Dimension 3100, Veeco, Plainview, NY, USA), Raman spectrometer with 532 nm excitation wavelength of He–Ne laser (Renishaw plc, Wotton-under-Edge, UK), and X-ray photoelectron spectroscopy (XPS, AXIS ULTR DLD, Kratos Analytical, Kyoto, Japan), respectively. The transmittance of the sample in the visible light region was determined by UV-Vis spectroscopy (Lambda 950, Perkin-Elmer, Waltham, MA, USA). The electrical signals of graphene biosensors for the detection of alkali ions were recorded by Hall effect measurement system (Hall 8800, Swin, Taiwan).

## 3. Results and Discussion

**Figure 1:**
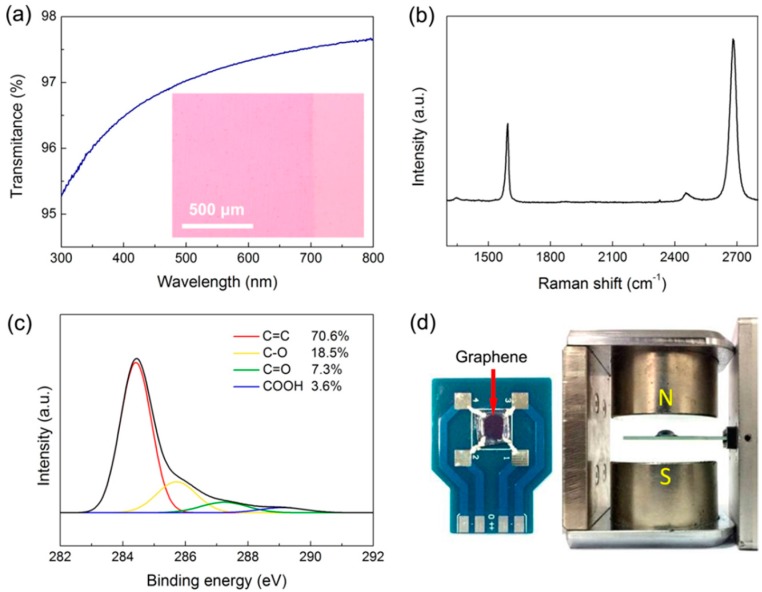
(**a**) The visible light transmittance of CVD graphene films. Inset: OM image; The corresponding (**b**) Raman and (**c**) high-resolution XPS C1s spectra; (**d**) Photograph of the graphene device measured based on the Van der Paul configuration.

The layer number and quality of graphene films were identified as presented in Figure 1a–c, because both of them significantly affected the performance of graphene-based biosensors [29]. In Figure 1a, the transmittance of the sample at 550 nm incident light is 97.2%, which is close to the theoretical value of single-layer graphene (97.7%) [30]. The OM image in the inset of Figure 1a shows that the contrast of a thin graphene layer placed on SiO_2_/Si substrate is very weak. The characteristic peaks of graphene can be found in its Raman spectrum (Figure 1b) with a sharp G-band (≈1580 cm^−1^) and a strong 2D-band (≈2700 cm^−1^) [31,32]. The negligible D-band (≈1350 cm^−1^), the high I_2D_/I_G_ ratio (1.98), and the narrow full-width at half-maximum of 2D-band (≈38 cm^−1^) demonstrate the nature of high-quality single-layer graphene prepared by the catalytic CVD method. The organic contaminants would be introduced to the graphene surface during the transfer process and also degrade sensor performance [23]. As the XPS results shown in Figure 1c, the high-resolution C1s spectrum of graphene can be fitted with four Gaussian peaks, consisting of C=C bond (≈284.4 eV), C–O bond (≈286.1 eV), C=O bond (≈287.1 eV), and COOH group (≈288.7 eV) [33,34]. The composition of oxygen-containing groups on the graphene surface was estimated to be 29.4%, which agreed with the value for graphene with a clean surface [35,36]. Figure 1d displays a photograph of the graphene Hall effect device and the setup of the electrical measurements based on the Van der Pauw method. The four silver paste electrodes were applied to the graphene device built on a printed circuit board. The electrodes were isolated from the buffer solution by coating with silicone (3140, Dow Corning, Midland, MI, USA), and a silicone reservoir was also built to hold the test solution.

**Figure 2:**
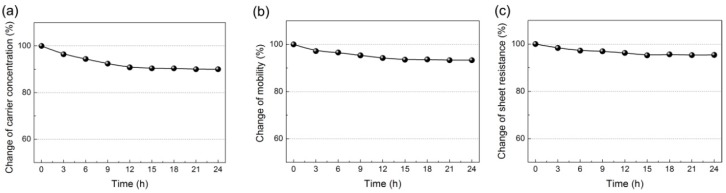
The changes of (**a**) carrier concentration; (**b**) mobility and (**c**) sheet resistance of graphene films as a function of the incubation time in DNA probe/1× TE buffer.

It is well known that the basal plane of graphene exhibits a strong interaction with foreign molecules, such as antigens, DNAs, proteins, etc., leading to the achievement of a very low detection limit of the biosensors based on graphene [37]. However, for biosensing applications, in most cases the graphene lacks target specificity without proper surface functionalization. Therefore, in order to efficiently and selectively capture K^+^ ions, a flexible single-stranded DNA with guanine-rich sequences (5′-GGTTGGTGTGGTTGG-3′) was immobilized on the graphene surface as a probe, which could fold into a tetraplex structure (guanine-quadruplexes) with K^+^ ions due to the formation of intramolecular hydrogen bonding [38]. Before performing the detection task, the graphene device was first incubated in 10 μM DNA probe/1× TE buffer solution (pH: 8). Figure 2 presents the change of the electrical properties of the device with the increase of incubation time in the probe solution. In Figure 2a,b, the decrease of carrier concentration and mobility can be attributed to the electronic *n*-doping and charged impurity scattering from the immobilized DNA strands, respectively [39,40,41]. In addition, the sheet resistance (*R*) is determined by the product of carrier concentration (*n*) and mobility (μ) based on the Van der Pauw principle: R ∝ 1/(μn) [42]. We found that the electrical properties remained almost unchanged when the sample was immersed in the probe solution for longer than 15 h, suggesting that the DNA adsorption on the graphene surface had reached the saturation state. After incubation for 15 h, the device could be ready for K^+^ ion detection without the interference from the background buffer ions and DNA probes.

**Figure 3:**
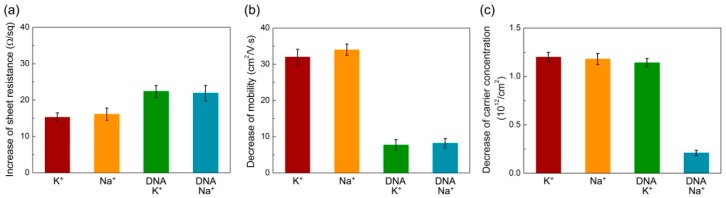
The variations of (**a**) sheet resistance; (**b**) mobility; and (**c**) carrier concentration of the graphene devices with and without DNA modification for distinguishing between K^+^ and Na^+^ ions.

In order to examine the K^+^ ion specificity of probe DNA-immobilized graphene device, a control experiment using Na^+^ ions was implemented and the sensor measurements using the graphene without functionalization were also performed for the comparison. The solutions containing K^+^ and Na^+^ ions with the desired concentrations were prepared by dissolving KCl and NaCl in 1× TE buffer, respectively. The device after incubation with DNA probes was rinsed with 1 mL 1× TE buffer for 3 times to remove weakly bound DNAs, and the electrical properties of graphene were recorded. The unfunctionalized graphene was also immersed in 1× TE buffer for 15 h, followed by the same rinsing process. A 100 μL drop with 1 nM K^+^ ions was individually placed on the surface of graphene with and without DNA immobilization for 6 h, and the electrical properties were recorded again after washing with 1× TE buffer. The standard deviation of each data point in Figure 3 is calculated from 8 samples. The same process was done using 10 nM Na^+^ ions to demonstrate the selective detection of our sensors. As shown in Figure 3a, the average sheet resistance increased the same amount for both ions when the experiments were conducted on pristine and functionalized graphene, respectively. Similar behavior can be seen from the decrease of carrier mobility in Figure 3b. We conclude that the change of sheet resistance and carrier mobility does not have the specificity of K^+^ ion recognition, even the graphene surface has been modified with guanine-rich DNA strand. In Figure 3c, the decreased amount of carrier concentration of the pristine graphene device for both ions is still similar. However, interestingly, Figure 3c shows an obvious difference of carrier concentration variation between the detection of K^+^ and Na^+^ ions in the case of probe DNA-immobilized graphene, suggesting that the monitoring of carrier concentration of the Hall effect device can effectively distinguish K^+^ ions from other alkali ions. 

**Figure 4:**
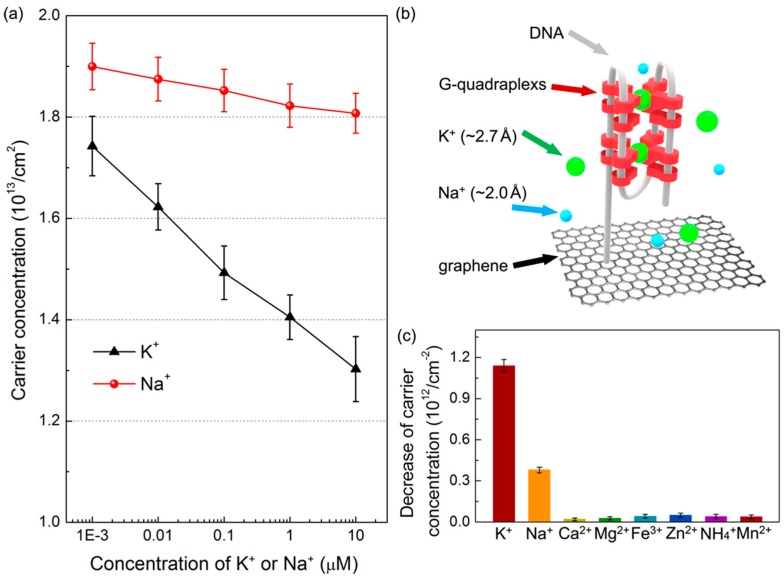
(**a**) Sensitivity and specificity comparison between K^+^ and Na^+^ ions; (**b**) Schematic illustration of the interaction between K^+^ ions and guanine-quadruplexes; (**c**) High selectivity for K^+^ ion detection over other interfering cations (added concentrations: K^+^ 1 nM; Others 10 nM).

The performance of graphene biosensors functionalized with DNA probes for selective K^+^ ion recognition was investigated, as presented in Figure 4a. The KCl or NaCl solution with 1× TE buffer was dropped on the device in sequence from 1 nM to 10 μM for reaction with DNA molecules. In this dynamic range (1 nM–10 μM ion concentrations), the carrier concentration linearly decreases 25.7% (from 1.75 × 10^13^ to 1.30 × 10^13^/cm^2^) when the K^+^ ion is the target. In contrast, it decreases only 4.7% (from 1.90 × 10^13^ to 1.81 × 10^13^/cm^2^) for sensing Na^+^ ions, demonstrating that our graphene sensing platform based on the Van der Pauw measurements provides a high sensitivity and distinct selectivity for the detection of K^+^ ions. Compared to other graphene-based devices [43,44,45], a lower detection limit (≈1 nM) can be achieved by our devices. For an example, the limit of detection for 10 nM K^+^ ions was reported using mechanically exfoliated graphene FETs [24]. The high specificity of our devices (≈4 times) can be attributed to the spatial compatibility between the central cavity of guanine-quadruplexes and the potassium ion radius, as schematically illustrated in Figure 4b. The interfacial space between two G-quadruplexes is in the range of 2.4–3.4 Å [46,47,48,49]. As this structure coordinates K^+^ ions (diameter: ≈2.7 Å), a bipyramidal antiprismatic arrangement can be implemented accordingly [48]. In order to further demonstrate the device availability in the complex situation, other cations commonly found in the human body were also employed to test our devices. In Figure 4c, obviously, the decreased amount of carrier concentration for K^+^ ions was much larger than other interference ions, even the added concentration of interference ions (10 nM) is 10 times higher than that of K^+^ ions (1 nM). The signal deviation for K^+^ ions is 3.2 and 16.3 times higher than that of Na^+^ and other cations (including Ca^2+^, Mg^2+^, Fe^3+^, Zn^2+^, NH_4_^+^, and Mn^2+^), respectively. The results confirm that the proposed graphene biosensor not only shows a high sensitivity, but also has a pronounced selectivity towards K^+^ ions.

## 4. Conclusions

In summary, the Hall effect biosensor fabricated with single-layer CVD graphene shows great sensitivity for highly selective detection of K^+^ ions. We demonstrated that among the electrical properties determined from Van der Pauw measurements, the change of carrier concentration of the device is extraordinarily sensitive to the added K^+^ ion concentration, and less dependent on that of Na^+^ ions. The K^+^ ion specificity is attributed to the spatial matching between K^+^ ions and guanine-quadruplexes, which is a tetraplex structure folded from guanine-rich DNA strands immobilized on the graphene surface. As a result, our devices exhibit a low detection limit (≈1 nM), wide dynamic range (1 nM–10 μM), and remarkable K^+^ ion selectivity against Na^+^ and other cations. The proposed sensing platform is feasible and effective in monitoring potassium ions in chemical and biological environments.
